# Automatic Classification and Severity Estimation of Ataxia From Finger Tapping Videos

**DOI:** 10.3389/fneur.2021.795258

**Published:** 2022-02-28

**Authors:** Adonay S. Nunes, Nataliia Kozhemiako, Christopher D. Stephen, Jeremy D. Schmahmann, Sheraz Khan, Anoopum S. Gupta

**Affiliations:** ^1^Department of Neurology, Massachusetts General Hospital and Harvard Medical School, Boston, MA, United States; ^2^Department of Psychiatry, Brigham and Women's Hospital and Harvard Medical School, Boston, MA, United States; ^3^Ataxia Center, Department of Neurology, Massachusetts General Hospital and Harvard Medical School, Boston, MA, United States; ^4^Movement Disorders Unit, Department of Neurology, Massachusetts General Hospital and Harvard Medical School, Boston, MA, United States; ^5^Athinoula A. Martinos Center for Biomedical Imaging, Department of Radiology, Massachusetts General Hospital and Harvard Medical School, Boston, MA, United States; ^6^Department of Radiology, Massachusetts General Hospital and Harvard Medical School, Boston, MA, United States

**Keywords:** ataxia, Parkinson's, machine learning, finger tapping, motor assessment, neurodegeneration, digital health

## Abstract

Digital assessments enable objective measurements of ataxia severity and provide informative features that expand upon the information obtained during a clinical examination. In this study, we demonstrate the feasibility of using finger tapping videos to distinguish participants with Ataxia (*N* = 169) from participants with parkinsonism (*N* = 78) and from controls (*N* = 58), and predict their upper extremity and overall disease severity. Features were extracted from the time series representing the distance between the index and thumb and its derivatives. Classification models in ataxia archived areas under the receiver-operating curve of around 0.91, and regression models estimating disease severity obtained correlation coefficients around *r* = 0.64. Classification and prediction model coefficients were examined and they not only were in accordance, but were in line with clinical observations of ataxia phenotypes where rate and rhythm are altered during upper extremity motor movement.

## Introduction

Cerebellar ataxia is a neurological phenotype caused by a wide range of diseases that affect the function of the cerebellum and lead to deficits in coordinated limb movements, gait and balance, speech, and eye movements. Clinical assessment of ataxia is typically conducted by visual inspection of the neurological exam and assigned a severity score using clinical rating scales such as the Brief Ataxia Rating Scale [BARS, (1)] and the Scale for the Assessment and Rating of Ataxia [SARA, (2)]. Leveraging technology to provide quantitative motor and cognitive assessments can be of great value for early diagnosis, tracking disease progression, and feature characterization of the disease (53). Although very useful, clinical rating scales rely on subjective human assessments that depend on the experience and perspective of the clinician and, by design, are relatively imprecise as evidenced by poor test-retest and interrater reliability (3–5). Digital assessments offer the ability to expand upon the information obtained by a clinician's examination. For example, quantitative assessments in pre-symptomatic or very early disease stages in neurodegenerative diseases can be sensitive in detecting early characteristics of the disease (6–9). Rather than discrete ordinal clinical scales that are performed during infrequent in-person clinical visits, digital assessments may provide much more frequent and precise measurements. This can result in greater statistical sensitivity and allow detection of smaller changes over time, leading the way toward improved outcome measures that can reduce the size and duration of clinical trials (10–14).

Previous studies in ataxia have used wearable sensors to quantify rhythmic finger tapping (15), limb movements such as Finger-to-Nose-Finger and Heel to Shin Test (16–18), free-living movement (19), and gait (20, 21). Other previously used instruments included smartphones to assess fine motor coordination skills (22) and computer mouse movement during web-based target clicking tasks (23) and free-viewing web searches (24) to assess upper-limb movements. Currently only one study applied computer vision and signal processing techniques on videos of arm movement assessments in ataxia to predict symptom severity (25). In parkinsonism, several studies have used video assessments for bradykinesia prediction (26), gait (27–29), and finger tapping (30, 31). See (32) for a comprehensive review.

In this study we used video recordings of a finger tapping task to assess the performance in classifying Ataxia or parkinsonism and in estimating the disease severity, as well as investigating the discriminative power of the features used in the models. Quantitative assessments based on video data have the advantage of scalability because cameras in phones and laptops could potentially be used to record the assessment, without requiring specialized or expensive equipment. Videos have the added advantage of directly capturing the position of body landmarks, whereas computing position from accelerometers in wearable sensors requires a double integration step that accumulates noise over time. In addition, video data can provide information about finger movement, which is more challenging using wearable sensors, which would require specialized sensors attached to the fingers or a glove embedded with sensors. We used the tapping signal obtained by the distance of the index and thumb fingers and computed its derivatives up to the 3rd order (i.e., jerk), as these kinematic features capture relevant aspects of the ataxia phenotype (33, 34). From the tapping and derivative signals, features were extracted based on clinical relevance such as the tapping period, variability of the tap period and amplitude, and the temporal slope of the tap amplitude (35–38). Features were grouped in five categories representing different aspects of the tapping behavior and Principal Component Analysis (PCA) was used to extract principal components of the feature types. Then, using the features extracted after processing the data, models were trained to predict the group class, either Ataxia, parkinsonism or controls (CTR), or to estimate symptom severity in Ataxia or parkinsonism.

## Methods

The study was approved by the Institutional Review Board at Massachusetts General Hospital and all the subjects provided written informed consent or assent. Individuals with ataxia and parkinsonism were recruited from Massachusetts General Hospital Ataxia Center and Movement Disorders Unit, and children with ataxia-telangiectasia were recruited in collaboration with the Ataxia-Telangiectasia Children's Project. Healthy control participants consisted of siblings of children with ataxia-telangiectasia and individuals recruited from Rally for Partners. A total of 301 videos from 191 unique participants performing finger tapping were assessed. These involved 169 videos from participants diagnosed with ataxia (128 participants with 41 repeated sessions), 74 videos from patients with parkinsonism or Parkinson's disease (61 participants and 13 repeated visits), and 58 videos from healthy control participants (50 participants and 8 repeated visits). Dataset demographics can be viewed in Table 1.

**Table 1:** Dataset demographics.

	**Ataxia**	**Parkinsonism**	**CTR**
N videos (female)	169 (80)	74 (22)	58 (26)
age: mean, std (range)	49 ± 22.4 (5–82)	67 ± 7.7 (45–85)	30 ± 20.2 (4–86)
Handness R/L count	152/17	70/4	52/6
bars arm R: mean, std (range)	1.15 ± 0.88 (0–3.5)	-	0
bars arm L: mean, std (range)	1.32 ± 0.89 (0–3.5)	-	0
bars_total: mean, std (range)	11.2 ± 0.5.6 (0–24)	-	0
UPDRS arm total L: mean, std (range)	-	3.68 ± 3 (0–12)	0
UPDRS arm total R: mean, std (range)	-	3.62 ± 2.94 (0–16)	0
UPDRS total: mean, std (range)	-	15.69 ± 9.69 (1–51)	0
Common arm score L: mean, std	0.33 ± 0.22	0.15 ± 0.13	0
Common arm score R : mean, std	0.29 ± 0.22	0.15 ± 0.12	0

### Clinical Assessment

All neurologic examinations were videotaped. Ataxia patients were scored on the Brief Ataxia Rating Scale (BARS) (range 0–30). Patients with parkinsonism were assessed with the Unified Parkinson's Disease Rating Scale (UPDRS) Part III Motor Examination (range 0–108) (39). Video data collection on the finger tapping task occurred immediately before or after the UPDRS was performed, ensuring that both the clinical assessment and task performance reflected the same state of the individual. Individuals took their prescribed medications as usual and no alterations were made for participation in the study.

For regression analysis, to allow comparison between Ataxia and parkinsonism patients, arm scores and total scores were normalized. For Ataxia, the common arm score was the BARS arm score, which is based on the finger to nose task, scaled in the unit range. A common arm severity score was calculated for the parkinsonism group by combining UPDRS tests involving the arms. The tasks used for this purpose included bradykinesia on three tasks. These subscores were summated to form a composite severity score (range 0–12) and scaled to unit range. CTR participants were assigned zero values for the common arm scores.

### Experimental Setup

**Figure 1:**
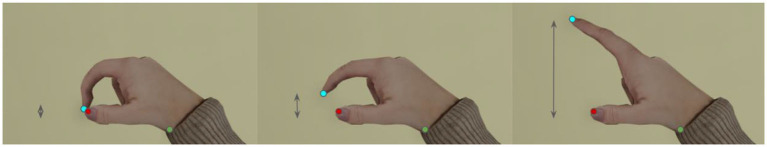
Finger tapping task. Participants were indicated to perform finger taping by extending the index finger from the thumb. The arrows indicate the distance between the index and thumb, and was used as a time series from which features were derived. The colored circles indicate the tip of the thumb (red) and index (blue) and the wrist (green).

Participants were seated in front of a tablet, which provided a video demonstration along with audio instructions for how to perform the task. The instructions were to perform finger tapping by touching the tip of the thumb with the tip of the finger as fast and fully as possible for 15 s, first using the dominant hand and then, once instructed, with the non-dominant hand. Participants were instructed to maintain a constant position of the hand during the finger tapping. If substantial movement was observed the participant was asked to repeat the task. Figure 1 illustrates the Finger Tapping task.

### Landmark Extraction

To extract landmarks of interest a residual convolutional network, namely, a ResNet152 (40), was used to identify the landmarks across frames. A model pre-trained on the MPII dataset to extract body landmarks was used (41) to leverage transfer learning and achieve faster training and higher performance (42, 43). The inputs to the model were frames of 960 x 540 pixels in batches of 1. Three landmarks of interest were extracted for each arm: the tip of the index, the tip of the thumb and the lateral side of the wrist. The DeepLabCut (DLC), (44, 45) toolbox was used in combination with in-house scripts (DeepNMA, https://github.com/neuropheno-org/DeepNMA).

The pipeline was as follows.

1. First, 80 videos were selected randomly and for each video, 20 frames were selected for manual labeling using a k-means clustering, as provided in DLC. The k-means clustering provided 20 frames that were the most different between each other. These frames were manually labeled.
2. The ResNet152 model was trained on the subset of frames to learn to identify the three hand landmarks. The training set was 80% of the data and the rest was used as a validation set to obtain the best performing model with the lowest mean absolute error (MAE).
3. After ~1.3M iterations, the retrained model was used to extract hand landmarks from the remainder of frames, generating a three-dimensional (thumb, index, lateral side of wrist) landmark time series for each hand-side.
4. Next, an iterative procedure was performed using DeepNMA to visualize and correct mislabeled landmark locations (further explained in the next section).
5. Once all the videos were inspected, the deep learning model was retrained with the relabeled data, and the landmark time series were re-inspected.
6. This procedure was repeated three times until the landmarks were correctly localized. A representative training and validation loss is presented in Supplementary Figure 1.

### DeepNMA

This package was created to visualize the landmark location time series, in the x and y directions, and its corresponding image frames, to preprocess the data and to classify groups and regress symptom severity. For quality control, DeepNMA was used to select the start and end of the finger tapping task for each arm. Outlier detection was performed using a process involving removing one sample at a time and re-estimating it with a cubic interpolation. The difference between the original value and the re-estimated value was used as a measure of outlier deviance. Missing samples, for a maximum consecutive period of 0.5 s, were interpolated with a cubic model. Then time series were visually inspected and, if necessary, time points were selected to plot the labeled video frame. In case of inaccurate labelings, the labels were corrected manually.

#### Signal Preprocessing

Some videos were recorded at 60 Hz and others at 30 Hz. Videos at the higher sampling rate were downsampled to 30 Hz. Then, the finger tapping amplitude time series (TS) was calculated by measuring the distance between the index and the thumb for each time point, creating a one-dimensional time series. To reduce possible high frequency noise resulting from frame-to-frame prediction jitter while preserving slower activity from finger movements, the TS was low pass filtered at 10 Hz using a FIR filter. Finally, given that amplitude of the finger tapping is dependent on the distance to the camera, signals were z-scored with a zero mean and unit variance to remove the effects of the camera distance.

#### Feature Extraction

A peak detection algorithm was used on the finger tapping amplitude time series (TS) to automatically detect peaks and troughs (46). Features were grouped in five categories: amplitude time series (TS) features, peak (Pk) and trough (Th) features with measures describing properties of the peaks and troughs separately, peak-to-trough (PkTh) representing the distance and amplitude between a peak and subsequent trough, and troughs-to-troughs (Th-Th) capturing the period between troughs. These categories represent meaningful components that capture the entire trajectory of TS—states where the index finger is tapping on the thumb (Th) and when the index finger is maximally extended (Pk), the trajectory to transition between states (PkTh), and the cycle from one tap to the other (Th-Th).

The measures captured by each feature group were as follows:

TS: The first three time derivatives (corresponding to velocity, acceleration, and jerk) were computed from TS. Using TS and each of the three derivatives, 11 measures were computed: mean, absolute mean, maximum, minimum, standard deviation, median, 10th and 90th percentile, range (maximum—minimum), interquartile range and entropy.

Pk and Th: Time series were generated by using the amplitude of peaks or troughs and computing their derivatives up to the 3rd order. The same 11 measures as for the TS features were extracted. In addition, the slope of a linear model, representing change over time of the peaks or troughs, was computed.

PkTh: The time and, separately the amplitude, between a peak and its subsequent trough was calculated and treated as a time series. The mean, standard deviation (std) and median of the time and the amplitude differences were extracted.

Th-Th: for each cycle from the trough to the next trough, the trajectory was parameterized with a quadratic model. The mean, std and median of the curvature coefficients of the quadratic models were extracted.

To reduce the dimensionality from 256 to 10, the Principal Component Analysis (PCA) was calculated for each of the five feature types and the first two Principal Components (PCs) were used as features for group classification and severity estimation.

#### Classification

**Figure 2:**
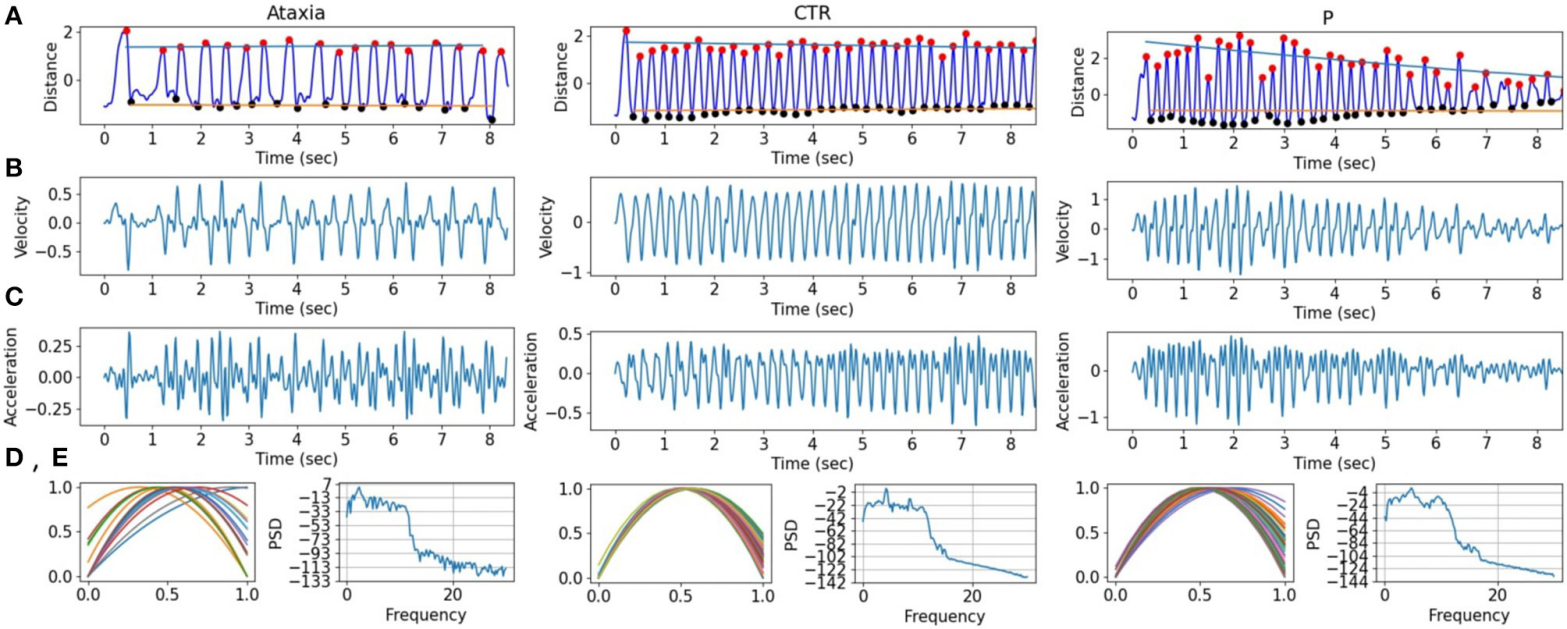
Finger tapping signals in ataxia, control (CTR), and parkinsonism (P) example subjects. **(A)** The finger tapping, measured as the normalized distance between the tip of the index and the thumb. The peaks (red) and troughs (black) are marked with dots, and the slope for the peaks and troughs are drawn in blue and orange, respectively. **(B,C)** First and second derivatives of the FT signal are shown. **(D)** The normalized quadratic modeling of the finger tapping is plotted for each tap. **(E)** The power spectrum density of the FT.

The first 2 PCs of the five feature types were used to classify ataxia vs. parkinsonism, ataxia vs. control (CTR), and parkinsonism vs. CTR. To address age differences in the groups, classification performance was assessed on ataxia vs. CTR under 45 years old, and ataxia vs. parkinsonism above 45 years old. In addition, to estimate the sensitivity in classifying mild ataxia, a mild-ataxia group was selected with a BARS arm score on the dominant arm < = 0.5 and classified against the CTR group. In Figure 2 some features are plotted from subjects representing their group.

Logistic regression with L1 or L2 penalty using the scikit package (47) was used and the model performance was tested with a 10-fold cross validation. To avoid overfitting, participants with repeated visits were always in the same fold when cross validation was performed. The regularization parameter was estimated in a non-nested 10-fold cross validation (48).

#### Regression

As in the classification, the first 2 PCs of the five feature types were used to predict behavioral scores. Three different models were employed to predict severity. One model was trained using the combination of all three groups together (ataxia, CTR, and PD), the second model was trained on only ataxia and CTR data and the third was trained on parkinsonism and CTR. Measures that we aimed to predict included common arm score from dominant and non-dominant sides, and Total BARS (for ataxia participants) and UPDRS (for parkinsonism participants). Ridge regression was used for prediction, and cross validation and hyperparameter tuning was performed as explained in the classification section.

## Results

### Disease Classification

Binary classification results between groups are shown in Table 2. Classification performance was high for ataxia vs. CTR (AUC 0.92) and ataxia vs. parkinsonism (AUC 0.91), but lower for parkinsonism vs. CTR (AUC 0.68). To assess the performance with mild-ataxia only participants, a model was trained against controls. The model performed well with an AUC of 0.72. As expected, performance decreased as differences with control participants would be smaller. In order to account for age differences, two models were trained: one including ataxia participants with age below 45 years vs. controls, and another including ataxia participants older than 45 against PD. Performance of the first models (ataxia age <45 vs. controls) had AUC of 0.93 while the second model (ataxia age > 45 vs. PD) AUC was 0.84.

**Table 2:** Group classification performance between ataxia, controls (CTR) and parkinsonism.

	**N**	**N %**	**AUC**	**Sens**	**Spec**
Ataxia vs. CTR	169, 58	0.75/0.25	0.92 (0.83–1.00)	0.89 (0.80–0.98)	0.81 (0.66–0.95)
Ataxia vs. parkinsonism	169, 74	0.66/0.33	0.91 (0.86–0.96)	0.94 (0.90–0.97)	0.72 (0.52–0.93)
parkinsonism vs. CTR	74, 58	0.66/0.33	0.68 (0.55–0.81)	0.69 (0.56–0.83)	0.64 (0.48–0.81)
Mild Ataxia vs. CTR	58, 58	0.50/0.50	0.76 (0.64–0.88)	0.70 (0.56–0.85)	0.83 (0.70–0.96)
Ataxia vs. CTR age <45	56, 45	0.55/0.45	0.93 (0.83–1.00)	0.88 (0.79–0.99)	0.85 (0.72–0.95)
Ataxia vs. parkinsonism age > 45	108, 73	0.60/0.40	0.84 (0.73–0.93)	0.79 (0.69–0.89	0.74 (0.66–0.95)

*The columns indicate the number of participants per group (N), the group ratio (N %), receiving operant area under the curve (AUC), sensitivity (Sens) and Specificity (Spec). For each measure the mean across cross-validation runs and the 95th confidence intervals are provided*.

**Figure 3:**
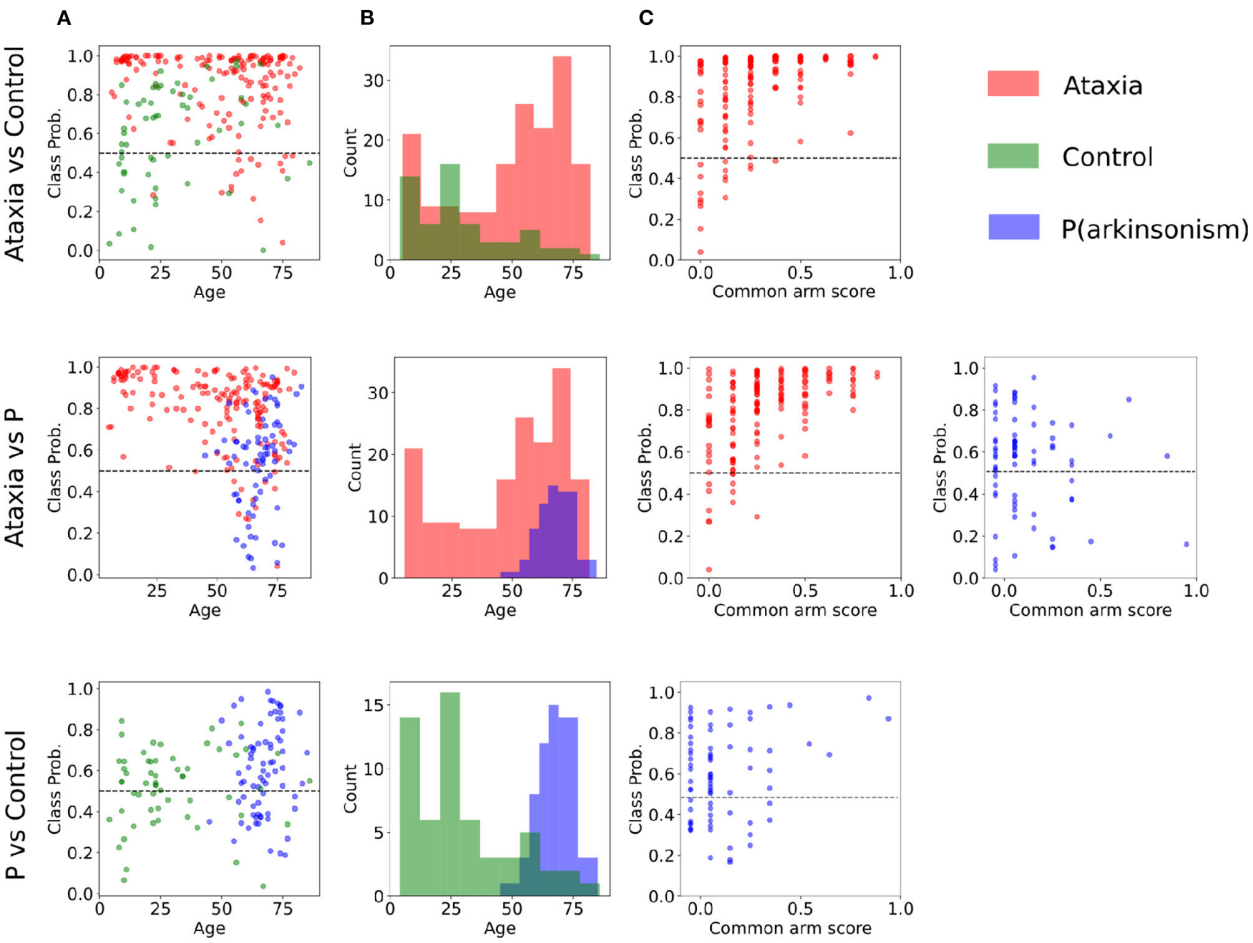
Classification probabilities and age distribution between pairs of groups. **(A)** Subjects' probability of the true class as plotted a function of age (incorrect predictions are below the dotted line). **(B)** Age distribution of each group. **(C)** Subjects' classification probability plotted as a function of symptom severity for the Ataxia and parkinsonism (P) groups.

To further evaluate how age and disease severity may affect model performance, correlation between classification probability were calculated. Only Ataxia's classification probability significantly correlated with age and common arm score. In the Ataxia vs. CTR, it correlated mildly with age (*r* = −0.18, *p*-value < 0.05) and with severity (*r* = 0.45, *p*-value < 0.001), similarly, in Ataxia vs. PD, age correlated significantly (*r* = −0.36, *p*-value < 0.05) as well as common arm score (*r* = 0.50, *p-*value < 0.001). The correlation between age and severity in the Ataxia group was *r* = −0.37, *p*-value > 0.001, thus severity might contribute to the age and classification probability correlation. In Figure 3, scatter plots represent the relationship between group classification probabilities and age and common arm score. Figure 3B shows the distributions of age within groups, likely contributing to the correlation between classification probabilities and age. In Supplementary Figure 2 the age distribution and scatter plots with classification probabilities and age or severity are plotted for the extended analysis with the mild-ataxia and for ataxia with bounded age ranges.

**Figure 4:**
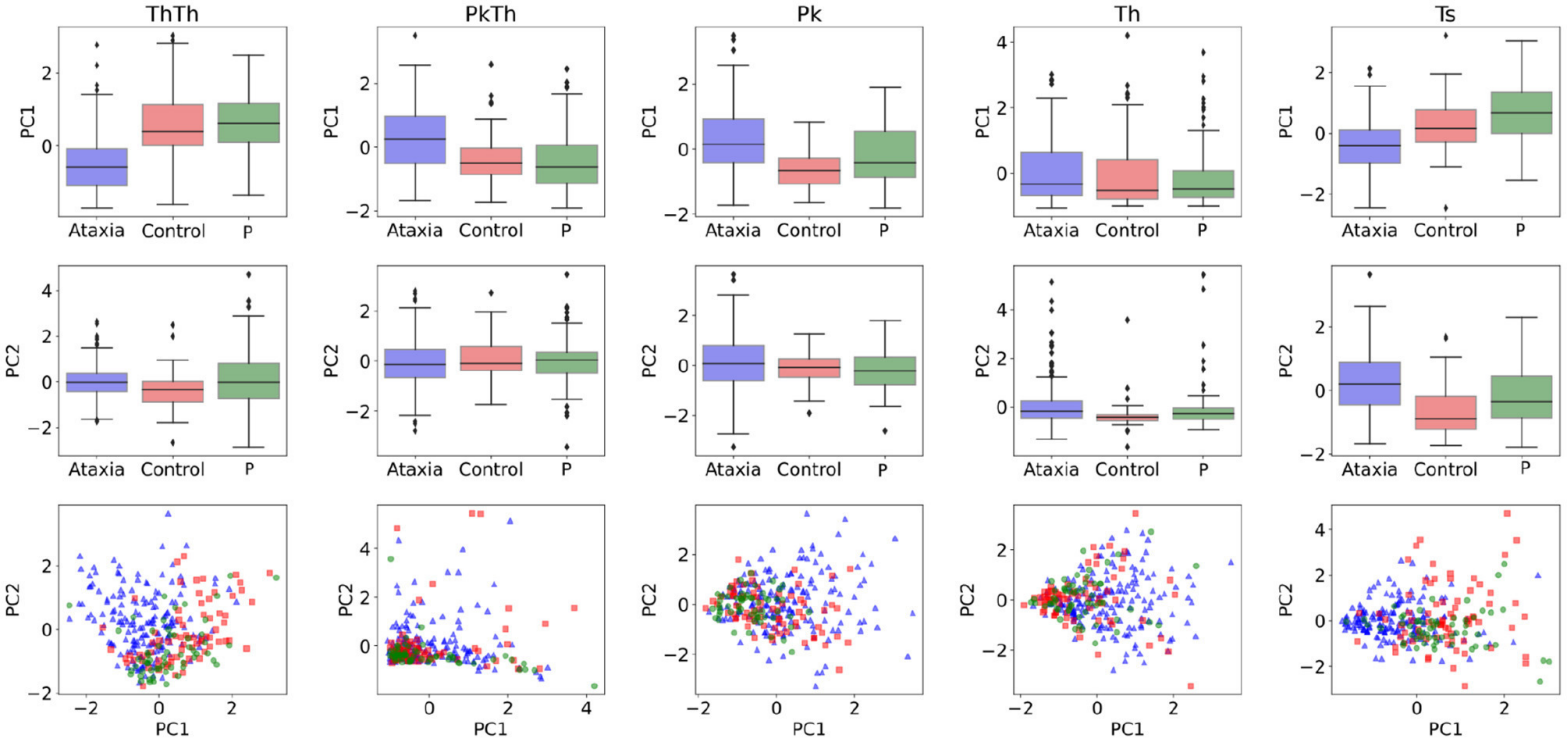
Feature characterization across ataxia, controls and parkinsonism (P). The first and second Principal Components (PCs) used as features for classification and symptom severity prediction are plotted as boxplots in the first and second row. The third row illustrates the 2d representation of the first two PCs per group.

In order to understand the general feature categories that contributed to model performance, on Figure 4 first two rows feature group box plots for the first and second PC representing the mean and quartiles of their distributions. Although the models are multivariate, individual visual inspection of the groups provide a sense of their discriminative power. This can be noted with the first PC of the Th-Th where the mean of the ataxia group is lower compared to the other groups. On Figure 4 row 3, the first two principal components are represented in a scatter plot. The separation between groups becomes more evident, with the ataxia group being spatially more separated than parkinsonism and controls, and explains the higher performance of the models in detecting ataxia.

**Figure 5:**
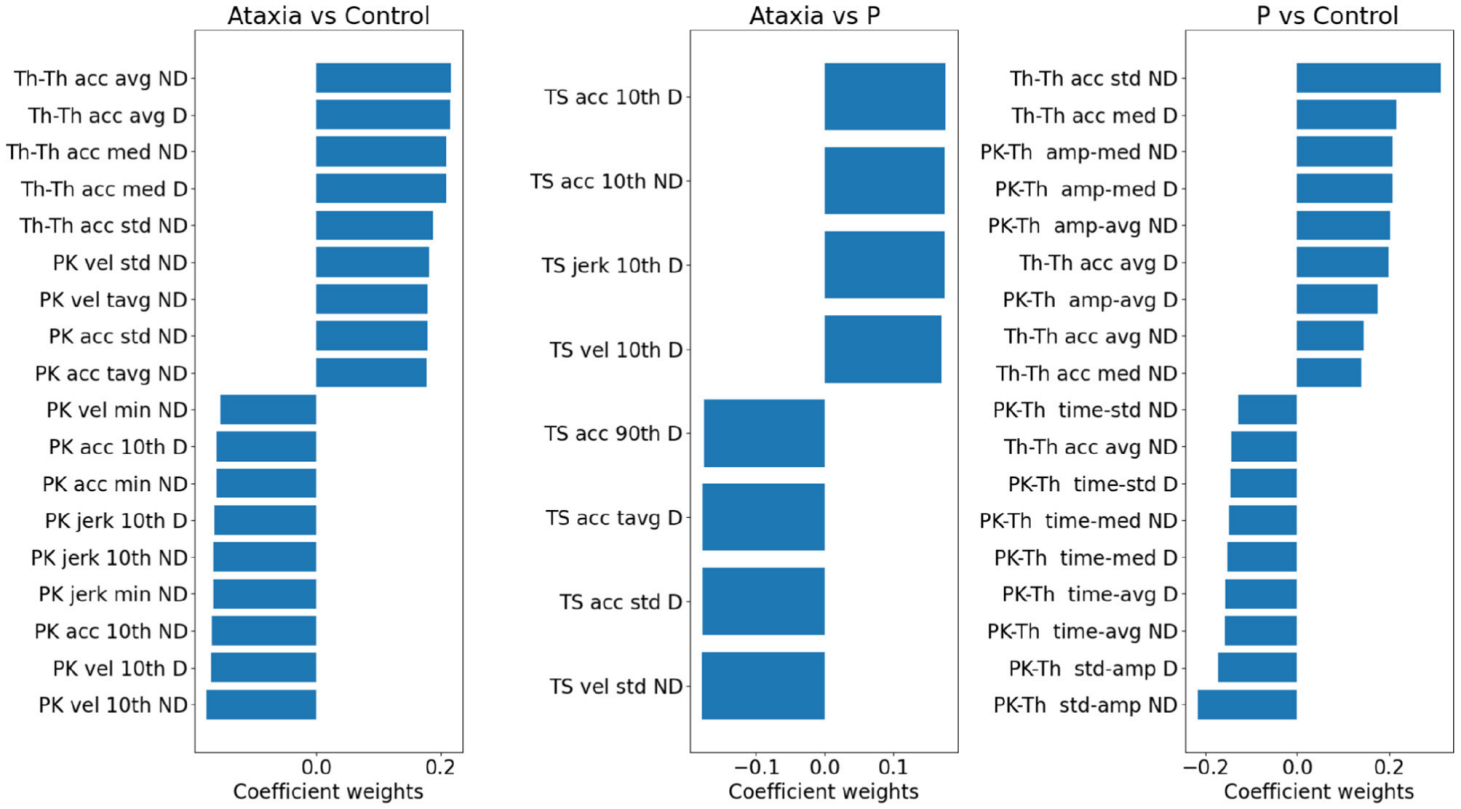
Model weights for group classification in ataxia, control and parkinsonism (P). For each pair of groups, a bar plot indicates the contribution of individual features in discriminating group classes. The bar plot values reflect the model weights of the PC features *individual weight of the PC. Positive weights denote higher feature values in the first group and vice versa. Only the largest 2% of the features are plotted. Due to the L1 penalty, some features were assigned zero weights and not plotted, hence for the Ataxia vs. parkinsonism the number of features are smaller than the other group pairs. vel, velocity; acc, acceleration; avg, average; tavg, total or absolute average; amp, amplitude; med, median; pth10, 10th percentile; pth90, 90th percentile.

To investigate the importance of individual features in separating group classes, the model weights representing the first two principal component contributions were multiplied by the PCA weights of the individual features, which indicate the features' contribution to the principal components. In Figure 5, the individual feature contributions to the model are represented for each group pair. The trough-to-trough (Th-Th) and peak (Pk) features contributed the most to discrimination between Ataxia vs. CTR. This can also be noted in Figure 3. Specifically, in the Th-Th, the average and std of the curvature coefficient (Th-Th acc) were larger in the ataxia group, indicating longer and more variable periods from tap to tap. With respect to the PK features, higher mean and std of velocity and acceleration (indicating higher peaks that change more over time), lower minimum values and 10th percentile were the most useful for correct classification of ataxia patients and controls. In contrast, classification of Ataxia against parkinsonism relied mostly on the TS features, with ataxia patients having larger 10th percentile and lower std. This indicated higher kurtosis for the Ataxia compared to parkinsonism but lower values compared to CTR, where the TS features distribution was less represented in the tails compared to the center. As can be seen, features with higher discriminatory power in one group might not be in another. In Supplementary Table 1, the T-scores of the most different measures between groups are reported for reference.

### Clinical Scores Prediction

**Figure 6:**
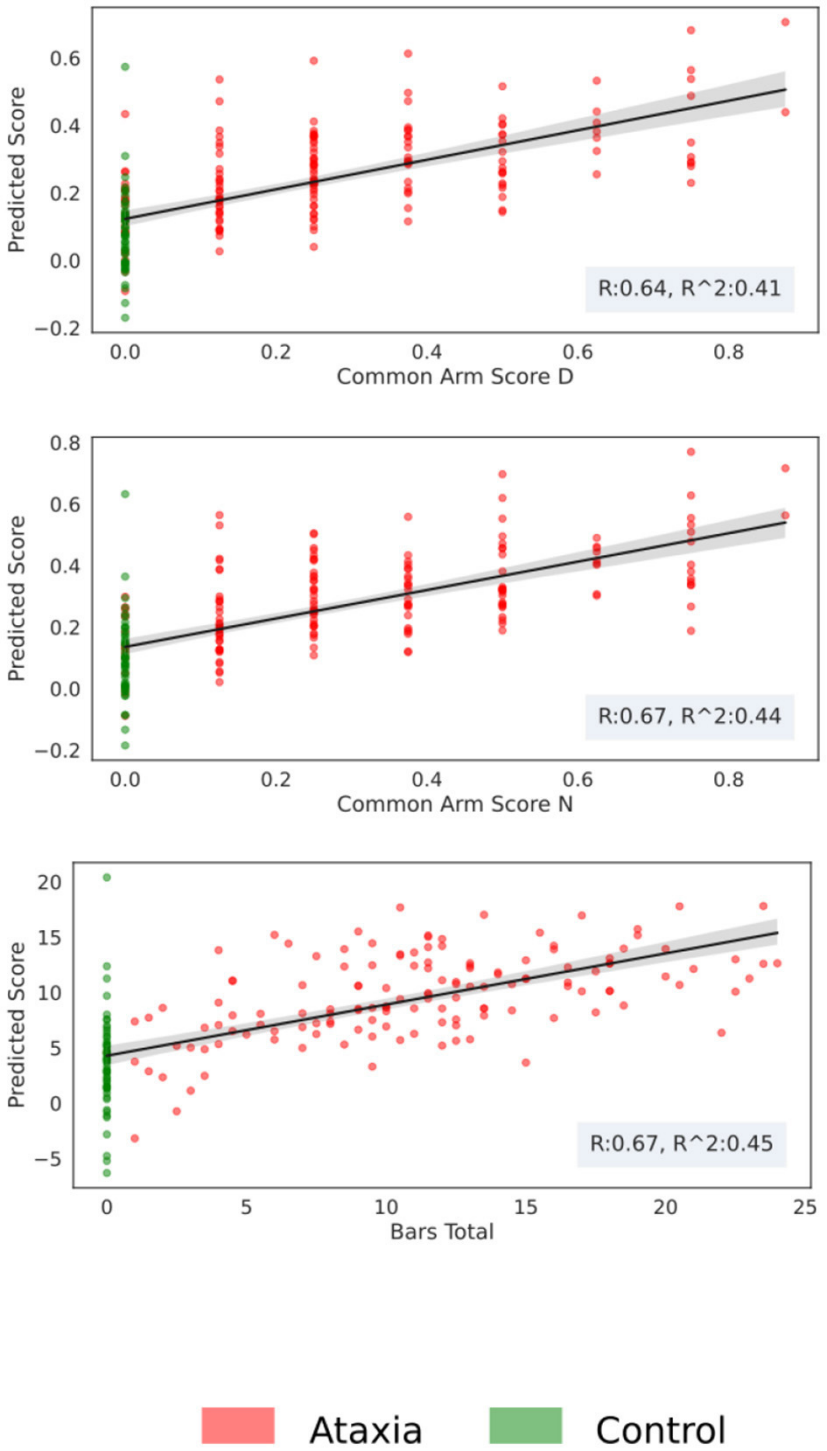
Score prediction with respect to the true clinical score for the Ataxia and CTR group. The scatter plot represents the estimated score vs. the real one, the shadow around the fitted line represents the bootstrapping 95th confidence intervals.

Using the first two principal components of the five feature types, models were trained to predict arm scores and total BARS or UPDRS. As shown in Table 3, the best performance was seen for the models trained on Ataxia and CTR data, with the non-dominant/dominant arm models achieving a correlation coefficient between predicted and actual scores of *r* = 0.67/0.64 and r2 explained variance of 44/41%. The score prediction performance with the Ataxia group only was considerably lower, with the non-dominant arm score *r* = 0.56, and r2 explained variance of 33%. The correlations between empirical and predicted scores can be seen in Figure 6. Performance of predicting parkinsonism scores was low, with the dominant arm score achieving the highest performance of *r* = 0.41 and *r2* = 0.17 in the parkinsonism and CTR groups and *r* = 0.21 and *r2* = 0.04 for the parkinsonism group only.

**Table 3:** Model performance in predicting symptom severity with different groups.

	**Common arm score D**		**Common arm score ND**		**BARS/UPDRS Total**	
	**r**	**r2**	**r**	**r2**	**r**	**r2**
Ataxia + CTR	0.64	0.41	0.67	0.44	0.64	0.41
Parkinsonism	0.41	0.17	0.29	0.08	0.43	0.18
+ CTR						
Ataxia	0.55	0.31	0.56	0.33	0.53	0.28
Parkinsonism	0.21	0.04	0.25	0.06	0.42	0.18

*The target measure is the estimated common arm score in the dominant (D) or non-dominant (N), and the BARS total in the Ataxia or UPDRS total in the Parkinsonism. As a performance measure, the correlation coefficient (r) and the coefficient of determination (r2)*.

**Figure 7:**
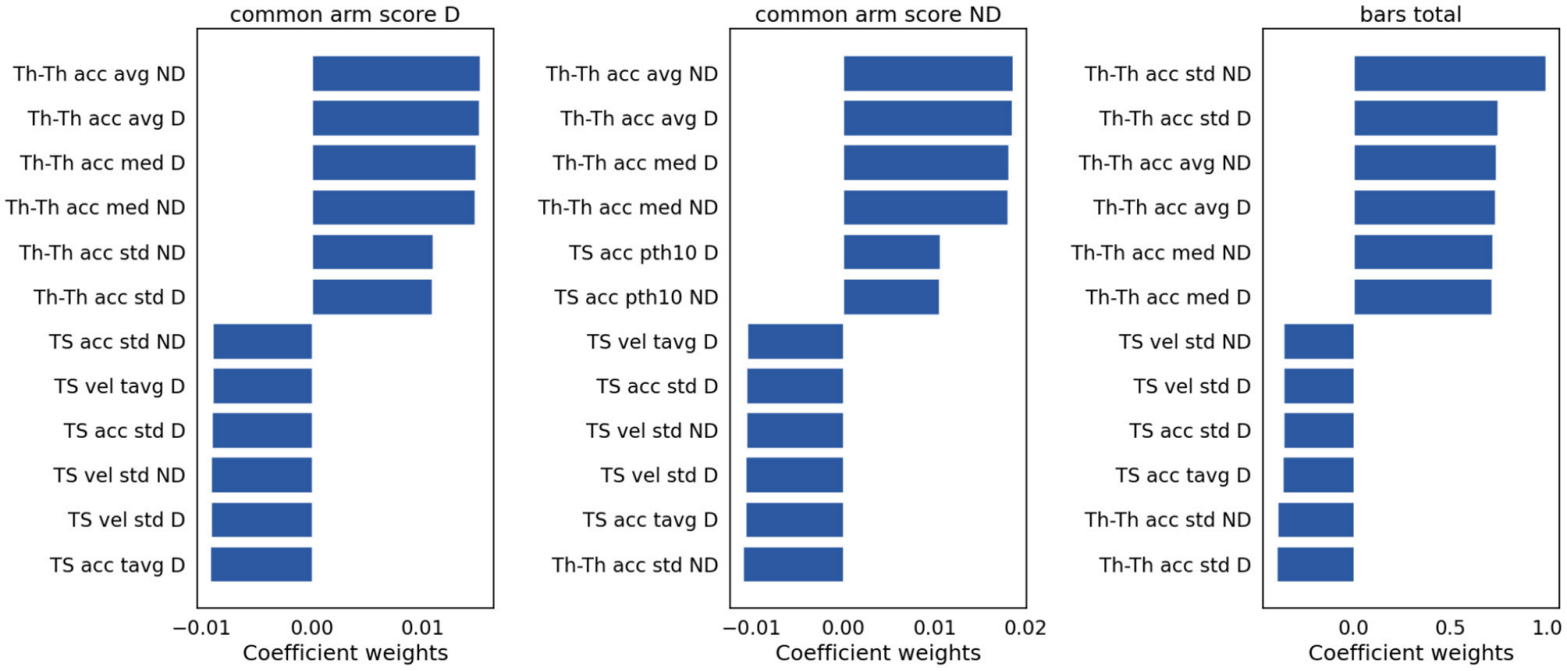
Model weights for clinical scores prediction. The bar plots indicate the magnitude and the sign of each feature contribution to predict the clinical scores. vel, velocity; acc, acceleration; avg, average; tavg, total or absolute average; med, median; pth10, 10th percentile.

To assess the contribution of individual features to the models explaining Ataxia severity scores, the product of the models weights and the PC weights were computed. Figure 7 illustrates the individual features contribution. As expected, the features with the highest representation in models trained to estimate common arm score dominant and non-dominant were similar. As in the classification between Ataxia and CTR, the Th-Th was the most contributing feature set. It represents the period from one trough to the other, i.e. from one index tap on the thumb to the next. The mean and std of the curvature parameter captured by the acceleration coefficient predicted Ataxia severity the most. In Figure 2 the FT curvatures from a representative subject for each group are illustrated. Higher mean indicates less concave curvature, i.e. flatter inverted U shape, and the std indicates that the period from trough to trough is more variable. The next feature type of most relevance was the TS. Especially, the total average and std of the TS velocity and acceleration. In Supplementary Table 2, the correlation of these features with Ataxia scores are reported.

## Discussion

Features extracted from video recordings during a finger tapping task can provide an objective measurement of upper extremity motor severity in Ataxia. In this study we demonstrate its effectiveness in classifying the ataxia phenotype against control and parkinsonism populations with an AUC > 0.90, and in quantifying motor function severity with a correlation of r > 0.6 between the predicted and true clinical scores. This approach can be useful for frequent and remote assessments and for tracking disease progression, as this type of video assessment is cost effective and can be performed at any desired frequency. This observer-independent analysis from features extracted after preprocessing converges on the clinical understanding, providing mutual support for the conclusion, and further evidence in support of the success of the method described: a single discrete movement as a reflection of the larger motor ataxia assessment and severity determination.

Sensitivity in detecting ataxia was above 0.90 with respect to controls and parkinsonism participants. As expected, sensitivity in detecting ataxia increased as ataxia severity increased (Figure 3C). There were substantial age differences between the three groups, however models performed well after restricting the age range to better age-match the groups (Table 3). The first principal component (PC) of the trough-to-trough (Th-Th) and peak (Pk) features was the most important for classifying Ataxia against controls. The time series (TS) features had the most contribution in discriminating Ataxia against parkinsonism participants. Based on the model's weights, the curvature of the quadratic model parametrizing the trough-to-trough (Th-Th) trajectories (plotted in Figure 2D), is wider and more variable in Ataxia compared to CTR. As reported in Supplementary Table 2, the median of the quadratic coefficients of the Th-Th was larger in Ataxia compared to CTR (*t* = 9, *p*-value > 0.001). The PK features capture position changes of the index finger when maximally extended before going back to tap the thumb. In Ataxia, compared to CTR, the mean and std of PK velocity and acceleration were higher, likely reflecting that the end positions of the index finger were more variable and erratic over time. When measuring statistical differences of individual features, reported in Supplementary Table 3, the individual PK features were not the most significant in the group of feature types. Instead, the power spectrum peak frequency was the most prominent, with ataxia having a lower peak frequency. Overall, the results are in accordance with clinical manifestations of ataxia characterized by impairment of rate, rhythm and force, and aligned with previous studies indicating higher spatiotemporal variability and slower finger tapping in individuals with ataxia (38, 49–52).

Performance in predicting severity scores in Ataxia was high, especially considering that finger tapping is only one of several tasks used by clinicians to assess upper extremity dysfunction. Models achieved good performance with correlation coefficients ranging 0.64–0.67, explaining 41–44% of the variance. BARS arm scores are discretized in relatively coarse intervals, whereas Total BARS is more continuous as it is an aggregation of multiple scores from the five BARS tasks. Model performance in predicting Total BARS and dominant arm scores were similar, indicating that discretization did not hinder performance and that the finger tapping features were capable of substantially predicting both arm and overall Ataxia severity. The main feature types that contributed in predicting severity scores were similar to the classification models, namely, the Th-Th and the TS features. This provides supporting evidence of the importance of these features for Ataxia diagnosis and severity estimation.

The video data in this study did not contain depth measurements, thus only two-dimensional landmarks were obtained. The absence of depth measurements in our study is a limitation. If during finger tapping participants moved the hand toward or away from the camera, in our 2D frame, it would represent a change of amplitude in the distance between the index and the thumb. The changes in amplitude of the tapping, thus, could be influenced by changes in the distance between the hand and the camera. That being said, participants were instructed to hold the hand in one position and not to move during the finger tapping assessment and if substantial movement was observed the participant was asked to repeat the task.

The approach employed in using video recordings to train machine learning models to detect ataxia and quantify the severity could help in tracking disease progression and make motor assessments more accessible to remote or resource-limited communities. The results indicate that this approach can accurately discriminate Ataxia from healthy individuals and from individuals with parkinsonism and can quantify upper limb and total disease severity. Future studies could benefit from combining video-based assessments across several motor tasks, which likely would lead to a more comprehensive phenotypic characterization with increased accuracy of classification and severity estimation.

## Data Availability Statement

The raw data supporting the conclusions of this article will be made available by the authors, without undue reservation.

## Ethics Statement

The studies involving human participants were reviewed and approved by Institutional Review Board at Massachusetts General Hospital. Written informed consent to participate in this study was provided by the participants' legal guardian/next of kin.

## Author Contributions

AN, SK, and AG contributed to the conception, design of the study, drafting the text and figures. CS, JS, and AG contributed to the acquisition of the data. AN, NK, SK, and AG contributed to the analysis of the data. All authors revised the manuscript for intellectual content.

## Funding

This work was supported by U.S. Department of Health & Human Services | NIH | Center for Scientific Review (NIH Center for Scientific Review) - 1R01EB0009048 [SK], DH | NIHR | Health Technology Assessment Programme (NIHR Health Technology Assessment Programme) - R01 NS117826 [AG] Ataxia-Telangiectasia Children's Project, Biogen Inc., [AG]. The authors declare that this study received funding from Biogen. The funder was not involved in the study design, collection, analysis, interpretation of data, the writing of this article or the decision to submit it for publication.

## Conflict of Interest

The authors declare that the research was conducted in the absence of any commercial or financial relationships that could be construed as a potential conflict of interest.

## Publisher's Note

All claims expressed in this article are solely those of the authors and do not necessarily represent those of their affiliated organizations, or those of the publisher, the editors and the reviewers. Any product that may be evaluated in this article, or claim that may be made by its manufacturer, is not guaranteed or endorsed by the publisher.
