# Supplementary material for: Automatic Classification and Severity Estimation of Ataxia From Finger Tapping Videos
Source: Front Neurol. 2022 Feb 28;12:795258. doi: 10.3389/fneur.2021.795258 (PMC8919801; doi:10.3389/fneur.2021.795258)
Supplement: Supplementary file 1 [file Table_1.DOCX]

**Supplementary Data: Automatic Classification and Severity Estimation of Ataxia From Finger Tapping Videos**


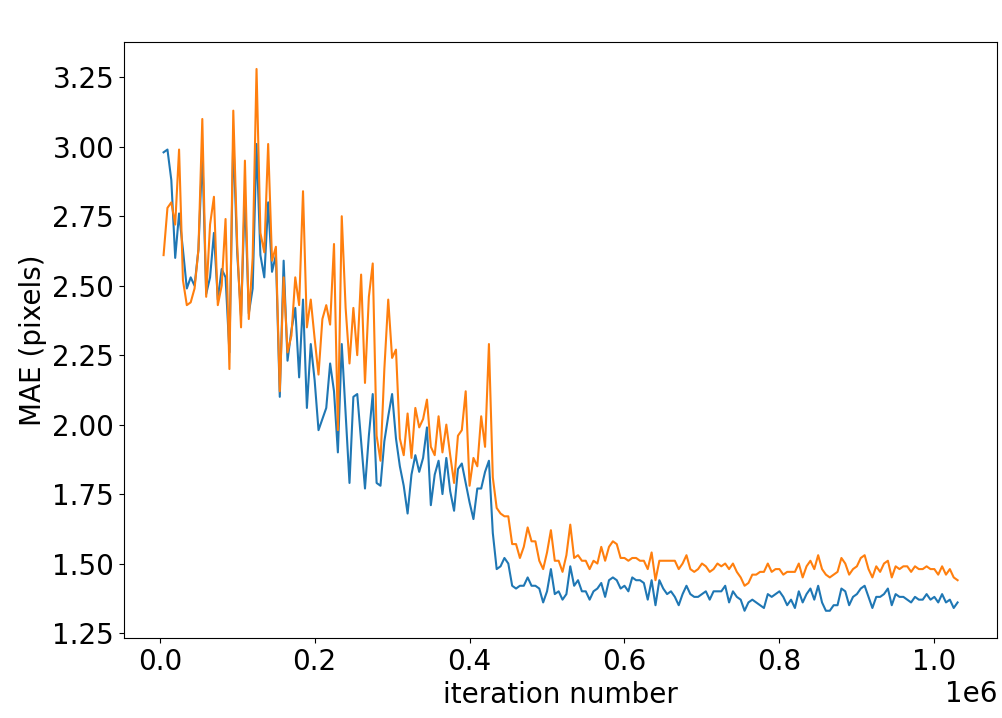


**Supplementary Figure 1.** Training and validation loss curvatures. The mean absolute error (MAE) across iteration epochs is represented in blue for the training and in orange for the validation.


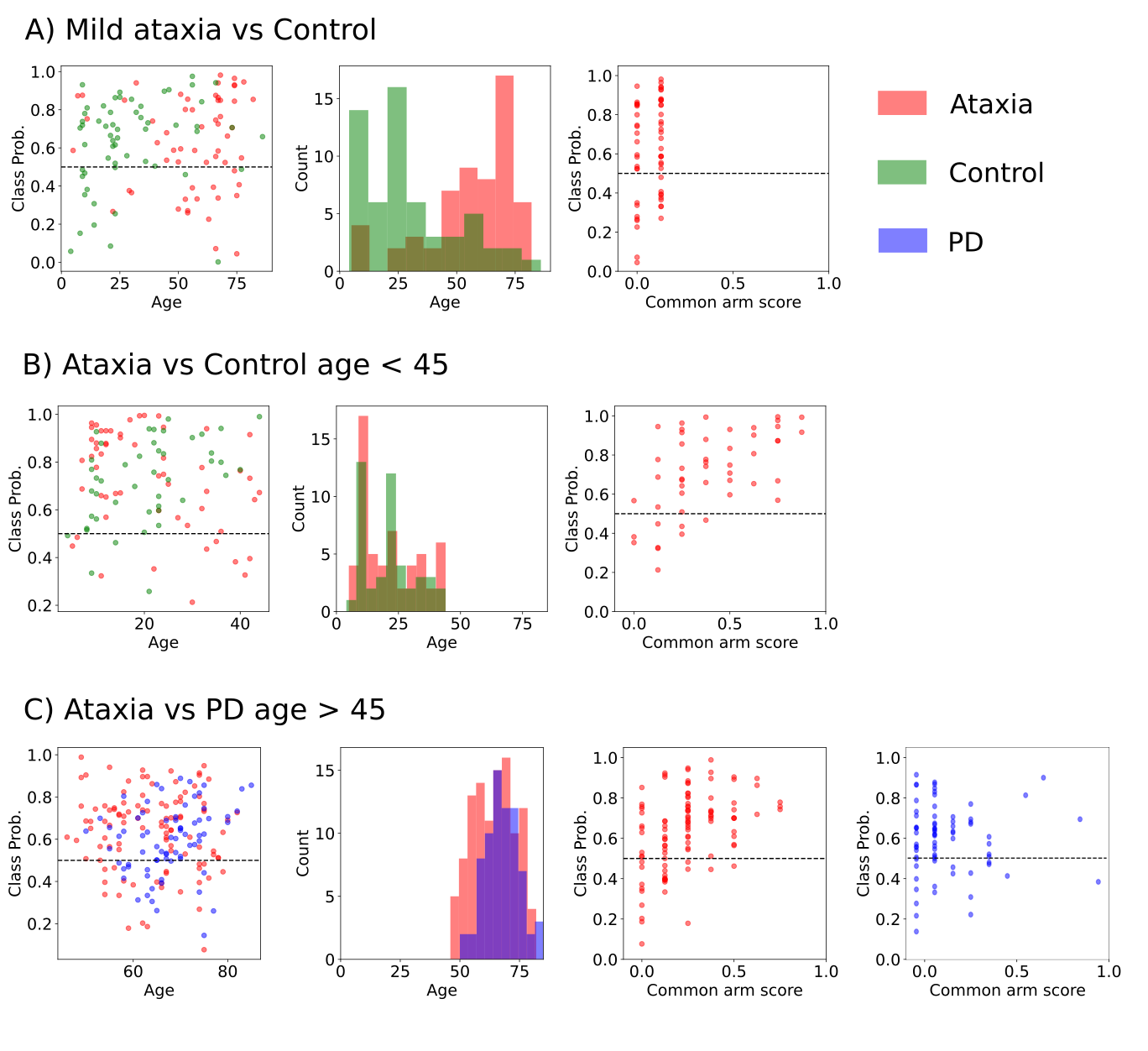
**Supplementary Figure 2.** *The subjects’ probability of the true class as a function of age are plotted in the first column (incorrect predictions are below the dotted line). On the second, the age distribution of each group is plotted, and on the third and fourth, the subjects’ classification probability are plotted as a function of symptom severity for the Ataxia and PD groups.*

| **Feature** | | | | **Ataxia - CTR** | |  | **Feature** | | | | **Ataxia - PD** | |  | **Feature** | | | | **PD – CTR** | |
| --- | --- | --- | --- | --- | --- | --- | --- | --- | --- | --- | --- | --- | --- | --- | --- | --- | --- | --- | --- |
| **Type** | **Order** | **Measure** | **Side** | **t** | **p** |  | **Type** | **Order** | **Measure** | **Side** | **t** | **p** |  | **Type** | **Order** | **Measure** | **Side** | **t** | **p** |
| ThTh | acc | med | D | 9 | 0.00 |  | Ts | acc | tavg | ND | -10.19 | 0.00 |  | Pk | jerk | tavg | D | 3.6 | 0.00 |
| Ts | acc | iqr | D | -8.88 | 0.00 |  | Ts | acc | std | ND | -10.08 | 0.00 |  | Pk | jerk | std | D | 3.54 | 0.00 |
| Ts | vel | iqr | D | -8.65 | 0.00 |  | Ts | vel | tavg | ND | -10 | 0.00 |  | Pk | acc | min | ND | -3.44 | 0.00 |
| ThTh | acc | avg | D | 8.47 | 0.00 |  | Ts | jerk | tavg | ND | -9.95 | 0.00 |  | Pk | acc | tavg | D | 3.37 | 0.00 |
| Ts | vel | tavg | D | -8.06 | 0.00 |  | Ts | jerk | pth90 | ND | -9.95 | 0.00 |  | Pk | jerk | std | ND | 3.36 | 0.00 |
| Pk |  | freq | ND | -7.98 | 0.00 |  | Ts | acc | iqr | ND | -9.94 | 0.00 |  | Pk | jerk | pth90 | ND | 3.36 | 0.00 |
| Ts | vel | pth10 | D | 7.97 | 0.00 |  | Ts | jerk | iqr | D | -9.92 | 0.00 |  | Pk | jerk | max | ND | 3.35 | 0.00 |
| Ts | jerk | iqr | D | -7.94 | 0.00 |  | Ts | jerk | std | ND | -9.91 | 0.00 |  | Pk | jerk | range | ND | 3.35 | 0.00 |
| ThTh | acc | med | ND | 7.88 | 0.00 |  | Ts | jerk | pth90 | D | -9.87 | 0.00 |  | Pk | jerk | iqr | D | 3.34 | 0.00 |
| Ts | acc | tavg | D | -7.83 | 0.00 |  | Ts | jerk | tavg | D | -9.87 | 0.00 |  | Pk | jerk | tavg | ND | 3.29 | 0.00 |
| Ts | vel | std | D | -7.48 | 0.00 |  | Ts | vel | std | ND | -9.77 | 0.00 |  | Pk | acc | std | D | 3.25 | 0.00 |
| Ts | vel | iqr | ND | -7.46 | 0.00 |  | Ts | jerk | iqr | ND | -9.74 | 0.00 |  | Pk | jerk | min | ND | -3.24 | 0.00 |
| Pk |  | freq | D | -7.43 | 0.00 |  | Ts | jerk | std | D | -9.73 | 0.00 |  | Pk | jerk | iqr | ND | 3.23 | 0.00 |
| ThTh | acc | avg | ND | 7.39 | 0.00 |  | Ts | vel | iqr | ND | -9.7 | 0.00 |  | Ts | jerk | std | ND | 3.22 | 0.00 |
| Ts | jerk | pth90 | D | -7.36 | 0.00 |  | Pk |  | freq | ND | -9.69 | 0.00 |  | Ts | acc | avg | ND | 3.2 | 0.00 |

**Supplementary Table 1. Individual features with highest t-scores between Ataxia, CTR and PD.** *Figure abbreviations: vel = velocity, acc = acceleration, avg = average, tavg= total or absolute average, amp = amplitude, med = median, pth10 = 10th percentile, pth90 = 90th percentile, iqr = interquartile range.*

|  | **Common arm score D** | **Common arm score ND** | **Bars total** |
| --- | --- | --- | --- |
| **TS vel tavg D** | -0.57 | -0.60 | -0.59 |
| **Th-Th acc med D** | 0.55 | 0.58 | 0.60 |
| **TS vel std D** | -0.55 | -0.59 | -0.56 |
| **Th-Th acc med ND** | 0.55 | 0.57 | 0.56 |
| **Th-Th acc avg D** | 0.53 | 0.57 | 0.59 |
| **TS acc tavg D** | -0.53 | -0.57 | -0.56 |
| **Th-Th acc avg ND** | 0.53 | 0.55 | 0.52 |
| **TS acc pth10 D** | 0.51 | 0.55 | 0.52 |
| **TS acc std D** | -0.50 | -0.53 | -0.53 |
| **TS vel std ND** | -0.56 | -0.60 | -0.50 |
| **TS acc pth10 ND** | 0.49 | 0.53 | 0.44 |
| **TS acc std ND** | -0.48 | -0.52 | -0.43 |
| **Th-Th acc std D** | N/S | -0.23 | -0.22 |
| **Th-Th acc std ND** | N/S | N/S | N/S |

**Supplementary** **Table 2. PC Correlation between clinical scores and PCs features and single measures.** *The Three most contributing PCs features are correlated with common arm scores. The most correlated single measure, from these three feature types, are presented. The feature types are the trough to trough, the time series (Ts) and peak (Pk) feature types, and the selected measures, the ThTh median acceleration, the Ts 10th percentile mean velocity, and the Pk maximal frequency from the power spectrum density. All measures were Bonferroni corrected with p-vals < 1e-14.*

|  | **common arm score D** | **common arm score ND** | **bars total** |
| --- | --- | --- | --- |
| **PK freq ND** | -0.63 | -0.65 | -0.63 |
| **TS vel pth10 D** | 0.61 | 0.63 | 0.59 |
| **TS vel tavg D** | -0.57 | -0.60 | 0.59 |
| **TS acc iqr D** | -0.57 | -0.60 | -0.59 |
| **TS vel std ND** | -0.56 | -0.60 | -0.56 |
| **TS vel iqr D** | -0.56 | -0.59 | -0.59 |
| **TS vel pth90 D** | -0.56 | -0.60 | -0.54 |
| **Th-Th acc med D** | 0.55 | 0.58 | 0.60 |

**Supplementary Table 3. PC Correlation between clinical scores and highest correlating features for Dominant and Non-Dominant arms.** *Figure abbreviations: vel = velocity, acc = acceleration, tavg= total or absolute average, med = median, pth10 = 10th percentile, pth90 = 90th percentile, iqr = interquartile range, std = standard deviation.*
